# Ashwagandha *(Withania somnifera)*—Current Research on the Health-Promoting Activities: A Narrative Review

**DOI:** 10.3390/pharmaceutics15041057

**Published:** 2023-03-24

**Authors:** Paulina Mikulska, Marta Malinowska, Miłosz Ignacyk, Paweł Szustowski, Joanna Nowak, Karolina Pesta, Monika Szeląg, Damian Szklanny, Eliza Judasz, Gabriela Kaczmarek, Ovinuchi Prince Ejiohuo, Magdalena Paczkowska-Walendowska, Anna Gościniak, Judyta Cielecka-Piontek

**Affiliations:** Department of Pharmacognosy, Faculty of Pharmacy, Poznań University of Medical Sciences, Rokietnicka 3, 60-806 Poznań, Polandovinuchi.ejiohuo@gmail.com (O.P.E.); mpaczkowska@ump.edu.pl (M.P.-W.); anna.gosciniak@student.ump.edu.pl (A.G.)

**Keywords:** Ashwagandha, *Withania somnifera*, winter cherry, herbal medicine, plant extract, antimicrobial activity, anticancer activity, anti-inflammatory activity

## Abstract

In recent years, there has been a significant surge in reports on the health-promoting benefits of winter cherry *(Withania somnifera),* also known as Ashwagandha. Its current research covers many aspects of human health, including neuroprotective, sedative and adaptogenic effects and effects on sleep. There are also reports of anti-inflammatory, antimicrobial, cardioprotective and anti-diabetic properties. Furthermore, there are reports of reproductive outcomes and tarcicidal hormone action. This growing body of research on Ashwagandha highlights its potential as a valuable natural remedy for many health concerns. This narrative review delves into the most recent findings and provides a comprehensive overview of the current understanding of ashwagandha’s potential uses and any known safety concerns and contraindications.

## 1. Introduction

**Figure 1:**
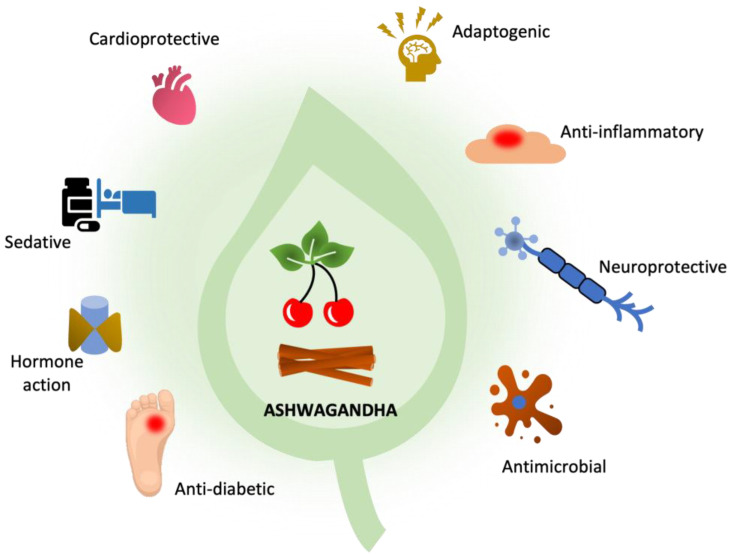
The comprehensive health benefit of Ashwagandha.

Indian ginseng is also known as Indian winter cherry, Ashwagandha, or the herb Vitania sluggard (*Withania somnifera*). The raw material used in medicine is the root, and the name “Ashwagandha” is derived from the word “ashwa”, meaning horse. It is believed that after consuming the root, one gains powers similar to that of a horse. The second part of the name “gandha,” means fragrance and refers to the characteristic smell of the fresh root of the plant [1]. Since ancient times, it has been traditionally used in Ayurvedic medicine as a substance that strengthens the nervous system. This is evidenced by its adaptogenic effects and medicinal uses—the so-called “rasayana”. Figure 1 below shows the comprehensive health benefit of Ashwagandha.

The history of its use in traditional Indian medicine dates back nearly 3000 years. Its root has been used as an aphrodisiac, narcotic, tonic, diuretic, anthelmintic and stimulant. It is naturally native to India, but it is also cultivated in other areas such as the Mediterranean countries, the Himalayan areas, Africa, Canary Islands, Cape of Good Hope and Australia [2,3,4].

In recent years, there has been a growing interest in the potential health benefits of Ashwagandha, particularly in the areas of stress management, cognitive function, and physical performance. Several studies have suggested that Ashwagandha supplementation may exhibit neuroprotective activity, be helpful in obsessive-compulsive disorder, and exhibit anti-inflammatory, immunomodulatory and antibacterial properties.

Additionally, there is evidence to suggest that Ashwagandha supplementation may be helpful in infertility, anticancer and antidiabetic treatment. Studies have suggested that Ashwagandha may exhibit cardioprotective properties, be helpful in the treatment of sleep disorders, improve stress resilience, reduce anxiety, be helpful in hypothyroidism, and enhance muscle strength and recovery.

While the potential health benefits of Ashwagandha are promising, further research is needed to fully understand its mechanisms of action and to determine its effectiveness in treating various health conditions. In this context, this paper aims to review the current literature on the activity of Ashwagandha, with a focus on its potential benefits for stress management, cognitive function, and physical performance.

## 2. Active Compounds

**Figure 2:**
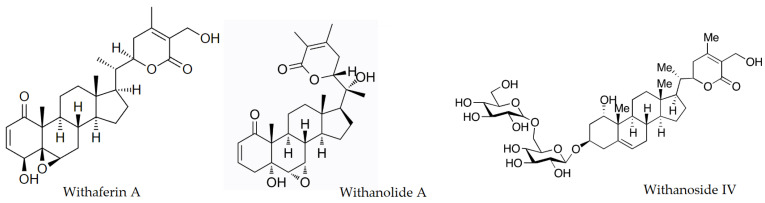
Chemical structures of the main active compounds present in Ashwagandha *(Withania somnifera)* root.

Ashwagandha is characterized by a rich phytochemical composition. Depending on the location of the raw material, it exhibits a diverse composition of chemical compounds. Its active substances that play a crucial role in pharmacological action are witanolides and alkaloids. Witanolides are compounds whose essential structure is that of ergostane, which has a six-membered lactone ring at the C-8 or C-9 position. The group of witanolides includes witanopherin A, witanolides A-Y, witanone, witadomniferin A, and witasomniferols A-C. Figure 2 shows the main active compounds present. Alkaloids include witanin, somniferin, somnin, tropin, somniferinin, pseudowitanin, pseudotropin, choline, kuskohigrin, isopeletierin, and anaferin [5]. Also present in the raw material are flavonoids which include 3-O-rutinoside, 6,8-dihydroxycemferol, quercetin and its glycosidic derivative, 3-O-rutinoside-7-O-glucoside.

Additionally, witanolid glycosides, which have a structure that contains a glucose moiety at position C-27, are also present in the raw material. This group of compounds includes sitoindoside IX and sitoindoside X. Ashwagandha also contains steroidal saponins that contain an acyl group–sitoindoside VII and VIII. Saponins, coumarins (scopoletin), sterols, chlorogenic acid, resins, lipids, carbohydrates and fatty acids have also been identified in the raw material [6].

## 3. Biological Activity

### 3.1. Neuroprotective and Anti-Neurodegenerative Effects

#### 3.1.1. Ashwagandha Use in Alzheimer’s Disease

For many years, the phenomenon of aging populations has been observed, which also implies a significant increase in the percentage of people suffering from dementia syndromes. Dementia is a syndrome with a multifactorial aetiology characterized by a range of symptoms caused by a brain disease, typically with a chronic and progressive course. This condition affects higher cortical functions, including memory, thinking abilities, orientation, comprehension, learning abilities, and emotional control.

Neurodegenerative diseases cause the destruction of the central nervous system, resulting in irreversible damage. Over the course of Alzheimer’s disease, an abnormal deposition of β-amyloid protein in the brain is observed. In its fibrillar form, it has a neurotoxic effect because it induces the formation of free radicals and impairs glucose transport in neurons, which leads to cell damage and death. Hyperphosphorylated τ proteins in Alzheimer’s disease form clusters surrounding the core of the senile plaque, consisting of β-amyloid. Physiologically, τ proteins stabilize microtubules, along with other proteins. Accumulating senile plaques are accompanied by microglia (inflammatory response cells), attempting to break down and remove damaged and dead neurons as well as senile plaques. Microglia cells produce toxins, destroying both diseased and healthy cells and enhancing the brain’s inflammatory response [7].

In studies conducted on human nerve cells, Ashwagandha has been shown to neutralize the toxic effects of β-amyloid, an implication in neurocognitive impairment during HIV infection [8]. A study was conducted on rats that were orally administered vitanon—an ingredient isolated from the root of *Whitania somnifera*. Significant improvements in cognitive function were observed as a result of the inhibition of amyloid β-42, and a reduction in pro-inflammatory cytokines TNF-α, IL-1β, IL-6, and MCP-1, nitric oxide, and lipid peroxidation was also observed. There was also a decrease in the activity of β and γ-secretase, enzymes responsible for the formation of insoluble neurotoxic aggregates of β-amyloid [9]. In addition, withaferin A extracted from Ashwagandha appears to be a promising ingredient in terms of Alzheimer’s disease treatment. It works by reducing β-amyloid aggregation and inhibiting τ protein accumulation. In addition, withaferin A is responsible for inhibiting oxidative and pro-inflammatory chemicals and regulating heat shock proteins (HSPs), the expression of which increases when cells are exposed to stressors. However, more medical studies are needed to assess the safety of withaferin A and to confirm its neuroprotective effects in the treatment of Alzheimer’s disease [10]. In addition, it has been noted that withaferin A from Ashwagandha extract significantly inhibits not only the production of amyloid β, but also the gene expression of neuroinflammatory molecules related to NF-κB [11]. A study was also conducted in transgenic mice that were given a half-purified extract of *Whitania somnifera* root containing mostly withanolides for 30 days. After this time, it was noted that Ashwagandha offset the negative effects of Alzheimer’s disease by increasing the number of the LDL receptor-related protein LRP1 (low density lipoprotein related protein 1) in the liver [12]. Increasing LRP1 levels reduced amyloid β and reversed the behavioural deficits in Alzheimer’s disease [13]. Studies suggest that LRP1 functionally modulates the steps required to form the β-amyloid precursor protein APP, which is critical for amyloid β production and APP processing [14]. The study also shows that LRP1 is a key regulator of protein proliferation τ [15]. The effect of *Withania somnifera* derivatives on the formation of β-amyloid 42 deposits in Alzheimer’s disease was studied. It was observed that withanolide A, withanolide B, witanoside IV and witanoside V interact with the hydrophobic core of β-amyloid 1–42 in the form of an oligomer, which prevents further interaction with monomers and reduces aggregation [16,17]. In another study, *Withania somnifera* extract was bioconverted by the fungus *Beauveria bassiana*. The resulting cysteine and glutathione derivatives of withaferin A were purified and fully characterized. It was shown that CR-777, a glutathione derivative of withaferin A, has neuroprotective effects and can be a protective agent against many neuronal stressors [18]. It is known that one of the most important components of Ashwagandha–withanolide A combats neurodegenerative processes in Alzheimer’s disease and Parkinson’s disease. However, it was only relatively recent that the ability of withanolide A to penetrate the blood-brain barrier (BBB) was demonstrated. In another study, adult mice were administered three different doses of vitanolide A intranasally: 1 mg/kg, 5 mg/kg, 10 mg/kg. The intranasal administration allowed for the penetration of the test substance into the cortex and cerebellum. After treatment with withanolide A, a significant reduction in cerebral infarction, improvement in blood-brain barrier function, and reduction in cerebral oedema were noted. An improvement in biochemical parameters and a reduction in excessively high levels of neurotransmitters, which had been caused by previous ischemia, were also noted. The highest dose administered (10 mg/kg) significantly reduced morphological damage, apoptosis and necrosis in brain tissues [19].

Neurodegenerative diseases also affect animals, not just humans, but in both cases the course of the disease and its pathomechanism is very similar. Canine Cognitive Dysfunction (CCD) is an age-dependent disease in which pathological changes in the brain are observed, leading to memory loss and impaired motor function. In both dogs and humans, a progression of oxidative damage in the brain is noted with age. In a study conducted on human embryonal neuroblastoma SK-N-SH cells, Ashwagandha extract was shown to have antioxidant properties (significantly reduces free radical generation). It also modulated cholinergic transmission, potentially inhibiting acetylcholinesterase activity, which may have benefits in the treatment of canine cognitive dysfunction and Alzheimer’s disease [20].

Additionally, it has been noted that withaferin A in the form of Ashwagandha extract significantly inhibits not only the production of amyloid β, but also the gene expression of neuroinflammatory molecules related to NF- κB [21].

#### 3.1.2. Ashwagandha Use in Parkinson’s Disease

In Parkinson’s disease, the degeneration of the dopaminergic neurons of the nigrostriatal system is observed. This leads to an imbalance between dopamine’s inhibitory action and acetylcholine and glutamic acid’s excitatory action. Factors that induce the degeneration of nigrostriatal cells include:

- Genetic conditions;
- Endo- and exogenous toxic factors;
- Neuroinfections;
- Oxidative stress;
- Reduced growth factors;
- The sum of the action of several of the above factors.

The disease is slightly more common in men than in women, and although the cause is not known, it is thought that this may be due to the protective role of estrogen [22].

A study was conducted on rats with 6-hydroxydopamine-induced Parkinson’s disease. Prior to an injection of 6-hydroxydopamine into the striatum, the rats were orally administered a *Withania somnifera* extract at doses of 100, 200, and 300 mg/kg body weight for 3 weeks. It was observed that administration of Ashwagandha significantly reduced lipoperoxidation, increased glutathione concentration, increased glutathione S-transferase, glutathione reductase, glutathione peroxidase, superoxide dismutase and catalase activities, catecholamines, and dopamine D2 receptor binding and enhanced tyrosine hydroxylase expression [23].

Although *Withania somnifera* significantly improves biochemical parameters in Parkinson’s disease, its effects depend on the dose administered [23,24,25]. In addition, in a study conducted on fruit flies, it was shown that administration of a standardized methanol extract of Ashwagandha root counteracts deficits associated with Parkinson’s disease [26]. In mice with Parkinson’s disease, administration of Ashwagandha extract not only improved biochemical parameters, but also reduced motor impairment compared to the control group [25,27]. It has been observed that oral administration of *Withania somnifera* extract (100 mg/kg, i.p.) to mice increases the levels of dopamine (DA), 3,4-dihydroxyphenylacetic acid (DOPAC) and homovanillic acid (HVA) and also normalizes the levels of lipoperoxidation markers in the striatum of the mice [25].

#### 3.1.3. Use of Ashwagandha in the Treatment of Huntington’s Disease

Huntington’s Disease is an incurable disease. Current medications only work on the symptoms and slow down the progression of the disease. The disease is inherited in an autosomal dominant manner, which means that statistically, half of the offspring will receive the disease-causing allele. A mutation in the IT15 gene encoding the huntingtin (htt) protein on chromosome 4 leads to a conformational change in huntingtin, into its insoluble form. The N-terminal part of the mutant huntingtin protein, containing expanded polyglutamine repeats, accumulates leading to accelerated neuronal apoptosis. This leads to an imbalance of dopamine, GABA, serotonin, and acetylcholine [28].

The compound 3-Nitropropionic acid (3-NP) is a potent neurotoxin. It induces oxidative and nitrosative stress, inhibits complex II of the mitochondrial electron transport chain (resulting in a deficit of moma energy), and is responsible for biochemical and neurobehavioural changes very similar to those observed in Huntington’s disease. In an animal model, symptoms of Huntington’s Disease were artificially induced by applying 3-NP intraperitoneally. It was observed that the chronic administration of Ashwagandha extract had a beneficial effect on biochemical parameters and motor function due to the antioxidant properties of the plant studied. There was a decrease in lipoperoxidation, a decrease in the levels of lactate and nitrate dehydrogenase, an increase in the levels of superoxide dismutase and catalase, and an unblocking of the mitochondrial complex and thus a restoration of ATP synthesis. The effect was dose-dependent—100 mg/kg and 200 mg/kg [29]. In another study in mice, the beneficial effect of withaferin A, isolated from Ashwagandha, was demonstrated. The inability of cells to maintain proteostasis is a sign of aging and a hallmark of many neurodegenerative diseases, including Huntington’s disease. In this mouse model, withaferin A ameliorates the impaired proteostasis by activating the heat shock response and delaying disease progression. The mice with Huntington’s Disease treated with withaferin A lived significantly longer, and restoration of behavioural and motor deficits was also observed, including a reduction in body weight. Biochemical studies confirmed the activation of heat shock, the reduction of mutant huntingtin aggregates, and the improvement of striatal function in the brain in mice. In addition, withaferin A significantly reduced inflammatory processes, as noted by reduced microglia activity [30,31].

### 3.2. Treatment of Obsessive-Compulsive Disorder, Alcohol Withdrawal Syndrome

Obsessive-compulsive disorder (OCD) is a chronic psychiatric disorder that involves patients experiencing symptoms in the form of intrusive thoughts and imagery. Patients perceive them as undesirable, unwanted, compulsive, and irrational. Although the severity of the cognitive disturbance can vary considerably from patient to patient, OCD makes daily life significantly more difficult—especially in its severe form, where it can significantly impair psychosocial functioning [32,33,34]. Genetic and psychological factors play a significant role in the aetiology of OCD, but structural and functional abnormalities within the central nervous system are equally important [33,34]. Obsessive-compulsive disorder is thought to be associated with the dysregulation of the serotonergic system [35]. Ashwagandha root extract may be a helpful adjunct to SSRIs in the treatment of patients with obsessive-compulsive disorder [35]. A study was conducted on mice that exhibited behavioural symptoms similar to those observed in OCD. In this animal model, mice were administered a methanolic extract of *Withania somnifera* (doses: 10, 25, 50, 100 mg/kg) and an aqueous extract of *Withania somnifera*. It was observed that the administration of the aqueous and methanolic extract of Ashwagandha significantly improved behavioural deficits in the mice, without affecting motor activity. The results obtained were similar to those of the standard treatments: fluoxetine, ritanserin, and para-chlorophenylalanine [36].

The effect of Ashwagandha in Alcohol Withdrawal Syndrome (AWS) in rats was studied. It was observed that the oral administration of Ashwagandha alleviated withdrawal anxiety due to chronic alcohol consumption, indicating a protective effect of the study plant in the management of ethanol-withdrawal reactions [37]. Haque et al., confirmed that Ashwagandha have beneficial effects on controlling behavioural changes, anxiety and seizures in alcohol withdrawal symptoms in rats, and it improves locomotor activity [38].

### 3.3. Anti-Inflammatory/Immunomodulatory Effects

Due to its properties, *Withania somnifera* is being studied for the treatment of many diseases associated with inflammation in the body, such as cardiovascular, pulmonary, and autoimmune diseases and diabetes, cancers, and neurodegenerative diseases. Preclinical studies have demonstrated the ability of this plant to regulate mitochondrial function and apoptosis and reduce inflammation by inhibiting inflammatory markers such as cytokines (including IL-6 and TNF-a), nitric oxide, and reactive oxygen species. Meanwhile, in a mouse model with lupus, a potential inhibitory effect of Ashwagandha root powder was demonstrated in conditions such as proteinuria and nephritis [39]. Ashwagandha is also being investigated for its efficacy in rheumatoid diseases. In a study conducted in an animal model, *Withania somnifera* root powder was administered orally to rats for three days, one hour before inflammation was induced by an injection of CFA (complete Freund’s adjuvant). In the control group (positive control), rats were administered phenylbutazone. Changes in the concentrations of a number of serum proteins, such as α2 glycoprotein, acute phase protein α1 and prealbumin, were demonstrated, along with a significant reduction in inflammation [40]. In a study using the HaCaT human keratinocyte cell line, an aqueous solution from Ashwagandha root was found to inhibit the NF-κB and MAPK (mitogen-activated protein kinase) pathways by decreasing the expression of pro-inflammatory cytokines, including interleukin (IL)-8, IL-6, tumour necrosis factor (TNF-α), IL-1β, and IL-12, and increasing the expression of anti-inflammatory cytokines. Based on these results, it can be concluded that the anti-inflammatory effects of Ashwagandha could potentially be used in the prevention of skin inflammation [41]. In a preclinical study of the anti-neuroinflammatory effects of Ashwagandha water extract (ASH-WEX) against lipopolysaccharide-induced systemic neuroinflammation, animals treated with ASH-WEX showed an inhibition of reactive gliosis; production of inflammatory cytokines such as TNF-α, IL-1β, and IL-6; and expression of nitro-oxidative stress enzymes. The underlying molecular mechanisms for the anti-inflammatory potential of ASH-WEX appear to involve inhibition of lipopolysaccharide (LPS)-activated NFκB, P38 and JNK/SAPK MAPK pathways. The results of this study suggest the potential use of *Withania somnifera* in suppressing nervous system inflammation associated with various neurological disorders [42]. Evidence presented in a study by Kanjilal et al. [43] showed that Ashwagandha extract applied for a period of 8 to 12 weeks can be useful in managing arthritis symptoms in patients. The immunomodulatory effect was confirmed in a study on the effect of *Withania somnifera* root powder on the stimulation of immune activity in immunodeficient mice. Administration of *Withania somnifera* was found to increase the total number of white blood cells and bone marrow cells, as well as to increase the titre of circulating antibodies and antibody-producing cells and to stimulate the production of immune cells and the phagocytosis of macrophages [44]. A randomized, double-blind, placebo-controlled trial with an open-label extension was conducted to evaluate the effect of Ashwagandha extract on the immune system of healthy participants. The results of the study showed that Ashwagandha extract significantly increased natural killer cell activity and cytokine levels, compared to placebo [45].

### 3.4. Antibacterial Properties

Drug resistance in micro-organisms is a major and growing threat, although now widely recognized. In recent years, a significant increase in infections caused by drug-resistant strains has become a major problem. It is known that the reckless and often unwarranted use of antibiotics has resulted in the development of drug-resistant strains, and in some situations, these drugs have become completely ineffective. Ashwagandha, therefore, appears to be a valuable addition to pharmacotherapy in the treatment of bacterial infections. Many of the drugs currently used to treat bacterial infections, despite their efficacy, have many dangerous side effects related to their toxicity. Ashwagandha is a safe, non-toxic plant with almost no side effects. In the studies that have been conducted, it has been proven to effectively inhibit the growth of methicillin-resistant *Staphylococcus aureus* and *Enterococcus* spp. [46]. *Withania somnifera* root extract has also been shown to effectively inhibit the growth of the Gram-negative bacteria *Escherichia coli*, *Proteus mirabilis*, *Pseudomonas aeruginosa*, *Salmonella typhi*, *Citrobacter freundi*, and *Klebsiella pneumoniae* [47,48,49]. The mechanism of its antimicrobial action is attributed to several of its properties; it has an immunomodulatory effect because it enhances immune reactivity (immunopotentiation), cytotoxic effects, and gene silencing [50].

Animal model studies also show that *Withania somnifera* is an effective treatment for salmonellosis, as it significantly alleviates the course of infection following infection with this pathogen [51]. *Withania somnifera* can also be an effective anti-caries agent, depending on the concentrations used. It significantly slows the growth of bacteria present in the oral cavity, such as *Streptococcus mutant* and *Streptococcus sobrinus*. It also inhibits bacterial acid production, acid tolerance, and biofilm proliferation [52]. It shows particular efficacy against *Salmonella typhi* [39]. The witanolides isolated from Ashwagandha induce cell death (acts on promastigotes) in *Leishamania donovani* by activating the process of apoptosis (ROS release from mitochondria occurs) and disrupting the mitochondrial membrane potential [53]. Studies have shown that Ashwagandha also exhibits valuable antifungal properties against some fungal species; it inhibits *Candida albicans*. It is important to note that *Aspergillus flavus* and *Aspergillus niger* seem to be resistant to the compounds it contains [48]. However, *Withania somnifera* glycoprotein isolated from its root tubers shows both antifungal properties against *Aspergillus flavus*, *Fusarium oxysporum*, *Fusarium verticilloides,* and antibacterial properties against *Clavibacter michiganensis* subsp. Michiganensis [54]. Murugan et al. [55] found that W. somnifera extract showed an enhanced antibacterial activity against *P. aeruginosa*. The study on the mechanism of antibacterial activity by Ashwagandha extract using morphological analysis and membrane stabilization assays showed that it acts by damaging the cell membrane of *P. aeruginosa*.

Studies in mice also show that *Withania somnifera* extracts (especially those at higher concentrations) are effective in the treatment of malaria, significantly reducing parasitaemia [56].

### 3.5. Support for Infertility Treatment

The definition of infertility given by the World Health Organisation (WHO) states that it is the inability to achieve pregnancy within 1 year, despite regular intercourse (3–4 times a week) and the lack of contraceptive use. According to estimates, this problem affects about 1.5 million couples in Poland, and recent years have seen an increase in the number of people seeking help. Infertility is a serious social, emotional, and demographic problem [57]. In men with oligospermia treated for 90 days with *Withania sommnifera*, an increase in sperm count, an increase in semen volume, and an increase in sperm motility were observed. Testosterone and lutenising hormone levels also increased, while PRL (prolactin) and FSH (folliculotropic hormone) levels were reduced. In studies conducted in men with normozoospermia, it was noted that the administration of Ashwagandha in the form of powdered root also significantly improved semen parameters. There was an increase in sperm count, improvement in sperm morphology, an increase in sperm volume, and an increase in sperm motility, which also increased the possibility of pregnancy in women. An improvement in the hormonal profile and an increase in antioxidant enzymes and antioxidant vitamins A, C, E in semen plasma was also observed [58,59,60]. No side effects were observed. In men taking the drug orally in the form of Ashwagandha root, not only were semen quality and reproductive hormone levels improved, but lipid peroxidation was also inhibited and CO (protein carbonyl groups) were reduced [58,61]. The presence of CO in blood and tissues is a reliable indicator of protein peroxidation. High levels of CO have been observed in the course of many diseases. To date, the exact relationship between CO groups, oxidative stress, and disease is not known. However, the use of CO groups as a biomarker is beneficial as these groups are relatively stable and appear relatively early [62]. In men with idiopathic infertility, it improves sperm parameters while having no side effects. In addition, Ashwagandha can also be considered as an alternative treatment instead of pentoxifylline [59]. Despite the very promising results, this topic requires further research due to insufficient data. The exact molecular mechanisms of action of *Withania sommnifera* and its active ingredients in the context of male infertility treatment are still unknown [63]. In one study, oral supplementation with high-concentration Ashwagandha root extract (HCARE) was shown to improve sexual function in healthy women. Compared to placebo, a significant improvement in sexual arousal, lubrication, orgasm, and an increase in the number of successful sexual intercourses was observed [64]. Chauhan et al., confirmed that compared to placebo, Ashwagandha root extract supplementation was associated with a statistically significant increase in the total DISF-M (derogatis interview for sexual functioning-male) scores [65].

### 3.6. Anticancer Effects

Cancer is a group of diseases in which cell division proceeds in an uncontrolled manner. This is due to mutations in gene-encoding proteins that are involved in the cell cycle, such as proto-oncogenes and anti-oncogenes. Statistics show that cancer is a serious and growing health and social problem. Despite worldwide research efforts in the fight against cancer, it remains a major cause of death.

Studies have shown that various compounds isolated from parts of Ashwagandha, such as the root, stem, and leaves, exhibit anti-cancer properties. Therefore, they can be used to treat cancer alone or in combination with other chemotherapeutic agents [66]. Witanolides are alkaloids present in the plant that show great anti-cancer potential. They are also the most promising compounds showing this action, as they play a major role in the induction of apoptosis. Ashwagandha is effective against cancers such as breast, colon, lung, prostate, and blood cancers [67]. It acts as a chemotherapeutic agent against many different types of breast cancer, especially ER/PR-positive breast cancer and triple-negative breast cancer [68]. In addition to its treatment, it also shows properties that prevent it. Research also suggests the potential of Ashwagandha in improving the quality of life of breast cancer patients [68]. According to research, withaferin A derived from Ashwagandha is also effective in the treatment of melanoma. This compound induces apoptosis and also reduces cell proliferation and inhibits melanoma cell migration [69]. The antitumor mechanisms of withaferin A in glioblastoma multiforme (GBM) were investigated. RNA-seq analysis, Western blot, immunofluorescence staining, qRT-PCR, and siRNA gene silencing were performed to determine the signaling pathways affected by withaferin A. It significantly inhibited GBM growth in vitro and in vivo and triggered intrinsic apoptosis of GBM cells. It arrested GBM cells in the G2/M phase of the cell cycle by defosphorylating Thr161 CDK1. This finding is important for the optimization of withaferin A-based regimens for the prevention and/or treatment of glioblastoma multiforme [70]. Jawarneh et al. [71] demonstrated that a combination of Ashwagandha extract and intermittent fasting has potential as an effective breast cancer treatment that may be used in conjunction with cisplatin. The combination was found to decrease cancer cell proliferation through apoptosis induction, while also reducing cisplatin-induced toxicity in the liver and kidney. Azab et al. [72] found that the extract had a protective effect against the harmful effects of radiation exposure, reducing oxidative stress and inflammation in the liver and spleen tissues. These findings suggest that Withania somnifera root extract may have potential therapeutic applications in protecting against radiotherapy-induced damage to vital organs such as the liver and spleen.

### 3.7. Antidiabetic Activity

The use of Ashwagandha is also considered in the context of anti-diabetic effects. However, there are relatively few reports on this issue. The antidiabetic properties of the raw material were interestingly described in a review paper by Durg et al. [63]. The results of the preclinical studies were promising. Animal studies have shown its ability to lower blood glucose levels [73,74,75,76,77]. In addition, Tekula et al. [78] confirmed that Withaferin A can effectively control induced type 1 diabetes in rats through modulation of Nrf2/NFκB signaling and therefore has significant potential for therapy. In silico studies have also confirmed withaferin A’s potential using molecular docking [79]. However, only one clinical study from the year 2000 showed a direct blood-glucose-lowering effect [80].

On the other hand, many studies have shown a beneficial effect on the lipidemic profile. In a study conducted on white albino rats with hypercholesterolemia, a reduction in cholesterol levels and also in the antioxidant effects of *Withania somnifera* were observed [76,77,81]. In the case of clinical trials on diabetes, despite not showing an effect on blood sugar levels, interesting results were achieved in improving the lipidemic profile, body weight, and blood pressure in a study by Agnihotri et al. [82]. Nayak et al. [83] showed an improvement in the lipidemic profile and patient assessment with the DDS17 scale assessing patients’ distress scale. Usharani et al. [84] noted that the administration of a standardised Ashwagandha extract under the name SENSORIL improved antioxidant parameters and the lipidemic profile and demonstrated the tolerability and safety of the raw material. Usharani, et al., in spite of its tolerability and safety, demonstrated an effect on lipidemic profile and a change in the reflection index [RI].

### 3.8. Cardioprotective Properties

The effect of Ashwagandha was studied in a group of albino rats in which myocardial necrosis was induced by isoprenaline treatment. A decrease in glutathione levels and a decrease in the activity of superoxide dismutase, catalase, creatinine phosphokinase, and lactate dehydrogenase were observed in the group of rats treated with *Withania Somnifera*. Lipid peroxidation levels also decreased significantly. These results indicate that *Withania somnifera* has a cardioprotective effect in an experimental model of isoprenaline-induced necrosis in rats [85]. Studies were also conducted on rats in which cardiac ischemia was induced. This caused significant myocardial necrosis, an oxidation–antioxidation imbalance, and an increase in lipoperoxidation. Histopathological studies have noted that the administration of *Withania somnifera* significantly reduces damage to the heart caused by ischemia. Ashwagandha has a cardioprotective effect due to its anti-apoptotic properties and due to its restoring of the oxidative balance [86]. The cardioprotective effect of withaferin A, a component of Ashwagandha known for its anticancer properties, was also studied. In this study in rats, low doses of withaferin A were shown to have a cardioprotective effect by upregulating the mitochondrial anti-apoptotic pathway due to an increase in AMP-activated protein kinase (AMPK) phosphorylation and an increase in the Bcl-2/Bax ratio (AMPK) [87]. This enzyme is involved in a number of processes responsible for maintaining energy homeostasis at both the cellular and whole-body level. AMPK regulates glucose, protein and fat levels in the nervous system and peripheral tissues and responds to hormonal signals by modulating food intake and energy consumption [88]. It is also known that AMPK is activated by caloric restriction and is involved in a number of processes correlated with ageing and diseases that often occur in older people. AMPK restores energy balance and is therefore thought to improve quality and length of life [88,89,90]. Surprisingly, however, only low doses of administered withaferin A (1 mg/kg) showed a cardioprotective effect. Administration of higher doses (5 mg/kg) was not effective [87].

### 3.9. Treatment of Sleep Disorders

Insomnia is defined as a situation in which the patient sleeps too little in relation to his or her needs, and this further leads to a deterioration in daytime functioning. Contrary to popular belief, insomnia is not just a condition in which nocturnal sleep is shortened, as the problem is much more complex and involves numerous complications. It is believed that insomnia can be related to difficulty initiating sleep, difficulty maintaining sleep or waking up too early. All of these symptoms can occur even when maintaining good sleep hygiene. The occurrence of these disorders leads to a poor sense of well-being, problems concentrating, emotional disturbances, cognitive disorders, and lack of motivation, which affects professional as well as social life. Researchers around the world agree that insomnia affects women more often than men. Old age is also a factor in insomnia. Unfortunately, there has been a significant increase in the proportion of people taking sleep medication, which indicates the scale of the insomnia problem. Nevertheless, it is believed that the condition is underdiagnosed. Epidemiological data differ by countries, which may be the reason for different methods of diagnosing this disorder. It is also worth noting that sleep constitutes approximately 30% of human life, hence the conclusion that the occurrence of any disorders in this area significantly disrupts the homeostasis of the organism [91].

Many current sleep medications have side effects, so it is thought that herbal remedies may provide an alternative therapy for insomnia. In one study, it was shown that the administration of Ashwagandha root extract to patients for 10 weeks (300 mg of the extract was administered twice daily) significantly improved the quality of sleep and also made it easier and faster to fall asleep [83]. Researchers believe that this could potentially be a treatment for people suffering from insomnia and anxiety. However, further research is needed. Studies have also been conducted in older people aged 65–80 years to assess the safety, efficacy, and tolerability of Ashwagandha root extract. Significant improvements in sleep quality, mental alertness upon awakening, and general well-being were observed. The tested treatment was found to be safe and effective, and the participants showed good tolerance [92]. Another study was also conducted to determine the effectiveness of the various compounds present in Ashwaganda for the treatment of insomnia. It was found that in mice, an alcoholic extract containing a high amount of active witanolides was not effective. An aqueous extract containing triethylene glycol as the main ingredient was therefore investigated. It turned out that this extract caused a significant induction of NREM (Non-Rapid Eye Movement) sleep. Commercially available triethylene glycol acted in the same way, although dose-dependently (in mice, 10–30 mg/animal was administered) [93].

NRS, or non-restorative sleep, does not have a standard and generally accepted definition, but it is considered by a large proportion of researchers to be a symptom of insomnia. It is usually defined as a subjective feeling of tiredness upon awakening, possibly due to insufficient sleep [94]. Researchers consider that more research is needed to define more precisely what NRS is [94,95]. As with insomnia, sickle cell disease (SCD) is much more common in women and in people with depression. It is estimated that SCD may affect around 10% of the world’s population and can occur independently without other insomnia symptoms [96,97,98]. A study was conducted to assess the effect of Ashwagandha on improving overall sleep quality in people with NRS. A group of healthy individuals exhibiting symptoms of NRS were administered 120 mg of standardised Ashwagandha extract (Shoden^®^) once daily for 6 weeks. It was observed that 72% of the subjects improved their sleep quality compared to 29% in the placebo group. In the treatment group, there was a significant improvement in SE sleep efficiency, sleep duration and total sleep time, as well as an improvement in WASO (wake after sleep onset). Significant improvements in physical, psychological, and environmental areas were also noted. No treatment-related side effects were reported throughout the course of the study [98].

There have also been studies evaluating the effectiveness of *Withania somnifera* in the context of treating sleep deprivation. Sleep deprivation has a huge impact on the functioning of not only the brain, but also the entire body. A number of studies show that it significantly impacts the deterioration of mood and cognitive and motor functions. It occurs when insufficient sleep leads to reduced efficiency, impaired alertness and a deterioration in overall health [99,100,101]. A study was conducted on adult male rats where the animals were subjected to sleep deprivation for one week. Indicators of oxidative stress were measured by spectrophotometry, while serotonin and dopamine levels were measured by ELISA. A reduction in antioxidant enzyme levels was observed in the sleep-deprived rat group. A significant reduction in the levels of free radicals and lipid peroxidation and an increase in the levels of antioxidant enzymes were observed in the group treated with *Withania somnifera* spice extract. Levels of dopamine and serotonin also increased compared to the untreated control group. It can therefore be concluded that *Withania somnifera* is an effective therapeutic agent for the treatment of sleep deprivation [102]. Good results in the treatment of sleep disorders have also been obtained by combining *Withania somnifera* root extract use and diazepam [103].

The findings of Baker et al. [104] suggest that Ashwagandha may have a positive impact on stress, sleep quality, energy levels, and mental clarity for college students. The study used qualitative analysis to assess the perceived impact of Ashwagandha on these factors, and the results indicated that participants who took Ashwagandha reported improvements in these areas compared to those who took a placebo. O’Connor et al. [105] conducted a double-blind randomized control trial that investigated the impact of Ashwagandha on stress, sleep quality, and food cravings in college students using quantitative analysis. The study found that Ashwagandha had a significant positive impact on reducing stress and improving sleep quality, but did not have a significant effect on food cravings.

### 3.10. Anxiolytic and Anti-Stress Effects

Stress is defined as the body’s biological response to external or internal stimuli. The compensatory response resulting from stress is called stress response and depends on the type of stress, its frequency, and its duration. It has been observed that stress as a factor disturbing homeostasis can not only exacerbate the symptoms of many diseases, but can also be their root cause. People who are under constant pressure at work or at home, and who feel overwhelming stress as a result, are much more likely to suffer from many diseases [106].

In the general population, anxiety disorders are the most common psychiatric problems. Unfortunately, due to their chronic nature and the compulsion to take multiple medications on a regular basis, some patients discontinue pharmacotherapy, which leads to relapses. The occurrence of numerous side effects, often unavoidable with conventional therapy, is cited as one of the reasons for discontinuing medication. Among other reasons, this is why it is so important to search for new therapeutics with fewer unwanted side effects. One study was conducted on a group of patients diagnosed with Generalized Anxiety Disorder (GAD). Participants were treated with SSRIs—Selective Serotonin Reuptake Inhibitors—and also took one capsule of Ashwagandha extract daily for six weeks. After the experiment, it was concluded that *Withania somnifera* extract could potentially support SSRI therapy in patients diagnosed with GAD syndrome [107].

It was also noted that Ashwagandha supplementation statistically and significantly reduced HAM-A (Hamilton Anxiety Rating Scale) and to a slightly lesser extent, reduced DASS-21 (Depression, Anxiety and Stress Scale). A reduction in morning cortisol and DHEA-S levels was also observed. In men, there was an increase in testosterone levels. In women, testosterone levels did not change. A significant reduction in PSS (perceived stress scale) scores was observed [103,108,109].

Ashwagandha’s anxiolytic effects may be due to several mechanisms. Firstly, Ashwagandha may decrease the activity of the hypothalamic–pituitary–adrenal (HPA) axis. In response to a stress stimulus, the HPA axis indirectly causes an increase in both cortisol and DHEA concentrations. DHEA (dehydroepiandrosterone) is a steroid hormone that is counted together with melatonin and growth hormone among the so-called ‘hormones of youth’, as their secretion decreases significantly with age [110]. The highest concentrations of DHEA occur between the ages of 20 and 30. In menopausal women, as well as in men during andropause, low levels of DHEA are the cause of numerous psycho-physical and psychosexual complaints. In addition, DHEA is a precursor to sex hormones and thus regulates their concentration. It has been noted that the administration of DHEA to both men and women can be an important factor in the treatment of sexual dysfunction occurring during menopause and andropause [111]. The correlation between stress and cortisol levels is well known. However, the link between stress and DHEA is less often reported. Although DHEA is an associated factor with health and longevity, excessive increases in DHEA concentrations indicate high exposure to stress or overactivity of the HPA axis. Elevated DHEA secretion has been observed in adults under acute stress and in individuals who have experienced traumatic events. Higher DHEA levels are also associated with cigarette smoking and alcohol consumption in middle-aged men. Ashwagandha’s anxiolytic effects are also associated with its anti-inflammatory and antioxidant effects. Under conditions of stress, depression and anxiety, inflammatory and oxidative processes are increased. Ashwagandha inhibits the above-mentioned processes by acting simultaneously on different mechanisms. Although they are discussed separately, the potency of Ashwagandha lies in the interaction between them, and is responsible for improving mood in people with depression [103]

A study was conducted using a sustained-release capsule containing Ashwagandha root extract (300 mg, Prolanza™). Participants took one Ashwagandha capsule daily for 90 consecutive days. It was noted that treatment with *Withania somnifera* once daily with one capsule significantly improved memory and attention, sleep quality, and overall psychological well-being. A reduction in stress levels was also noted. The treatment appeared to be safe and well tolerated [112]. A study was conducted in female rats to determine the effects of acute sleep deprivation on immune function and the modulation of this state by the application of an aqueous extract of *Withania somnifera* leaves. An increased expression of pro-inflammatory and immune factors was observed in the sleep-deprived animals (GFAP, TNF α, IL-6, OX-18, OX-42). In the Ashwagandha-treated group, there was an inhibition of stress-induced apoptosis with an increase in the expression levels of NF-κB, AP-1, Bcl-Xl and cytochrome c [113]. A group of patients with schizophrenia, depression, and anxiety disorders were also studied. Participants were treated with standardised *Withania somnifera* extract. The results of the study suggest that the extract has promising effects in the treatment of patients with depression, anxiety disorders, and schizophrenia. The exact mechanism of action is, however, still not known [114]. Clinical and experimental studies have confirmed the efficacy of Ashwagandha in the treatment of anxiety disorders. However, it is only relatively recently that one of the components responsible for the anxiolytic action of Ashwagandha was identified. The component identified is ferulate doconasil—DF (DF doconasyl ferulate), an alkyl ester of long-chain ferulic acid. Upon completion of the study, it was noted that, as with diazepam, DF exhibits an anxiolytic effect that is blocked by flumazenil. DF acts by modifying the activity of the GABA-A receptor complex [115]. Side effects typical of benzodiazepines, such as impairment of cognitive and motor functions, were not observed during the course of the experiments [109,115,116].

Research has shown the beneficial effects of *Withania somnifera* on brain neurotransmitter levels (increased GABA and decreased dopamine) in alcohol-dependent rats [117]. Ashwagandha also has potential in the adjunctive treatment of exacerbations of schizophrenia symptoms. Administration of *Withania somnifera* extract to patients resulted in a reduction in negative, general, and total PANSS (Positive and Negative Syndrome Scale) symptoms compared to placebo. There was a significant improvement in PPS (Perceived Stress Scale) scores. These significant improvements were noted in the study group, and they experienced only minimal side effects [118]. Remenapp et al. [119] found that the supplementation had a positive effect on cognitive function, specifically on attention and working memory, as well as on mood, reducing symptoms of anxiety and stress. The findings suggest that Withania somnifera may have potential as a natural supplement to enhance cognitive function and improve mood in adults.

### 3.11. Adaptogenic Effect

Adaptogens are herbs that improve an individual’s ability to cope with stress and adapt to change. The most recent definition of an adaptogen is “a class of metabolic regulators that enhances the body’s ability to adapt to environmental factors and avoid the damage they could imply.”

The ideal adaptogen should reduce negative changes caused by stress, be safe and act beneficially even when the dose given is higher than required, and be free of adverse side effects, such as not affecting the functioning of the body more than needed [120]. Based on the above-mentioned characteristics, Ashwagandha can be considered as an adaptogen.

A study was conducted on a group of horses given Ashwagandha root extract. The animals were subjected to various stressors, such as heavy exercise, separation, and noise. Haematological, biochemical, hormonal, and immunological parameters were studied during the experiment. After 21 days, a statistically significant decrease in cortisol, epinephrine, glucose, triglycerides, creatinine, IL-6, alanine aminotransferase, and aspartate aminotransferase was observed in the treated group. This indicates the adaptogenic, antioxidant, and immunostimulating effects of Ashwagandha. The adaptogenic effects of the standardised extract of *Withania somnifera* root and *Panax ginseng* were also studied in rats subjected to chronic stress (CS) using the Footshock method. Chronic stress induced the induction of hyperglycaemia, glucose intolerance, elevated plasma corticosterone levels, increased gastric ulcers, sexual dysfunction, cognitive deficits, immunosuppression, and mental depression. The entire range of the aforementioned disorders was significantly alleviated by the administration of *Withania somnifera* extract and *Panax ginseng* prior to the stressor [121]. The effect of an aqueous fraction devoid of witanolides, which was isolated from the root of Ashwagandha, was also studied. The study investigated the adaptogenic activity of a novel withanolide-free aqueous fraction from the roots of Withania somnifera in rats and found that it exhibited significant anti-stress effects, including improved swimming endurance and reduced adrenal gland weight, without causing any adverse effects. [122].

### 3.12. Treatment of Hypothyroidism

Thyroid diseases are among the many conditions that pose significant problems in the 21st century. Hypothyroidism is the most common in clinical practice. Ashwagandha extract lowers thyroid hormone levels in the blood and regulates glucose metabolism, which is impaired in thyroid disease.

Ashwagandha is one of the few medicinal plants that is free of iodine. Iodine is what stimulates the thyroid gland to produce the hormones T3, T4, and TSH. However, Ashwagandha has been shown to be more effective in the treatment of subclinical hypothyroidism than in advanced hypothyroidism. Despite this, it can be used as an adjunct in conventional therapy [123]. The substance that mainly stimulates thyroid activity is withaferin A, which also has anticancer effects. The antioxidant effect of withaferin A normalises the function of the thyroid gland [124,125]. A study designed to evaluate the efficacy and safety of Ashwagandha root extract in patients with subclinical hypothyroidism was conducted. A total of 50 subjects with elevated serum thyrotropic hormone (TSH) levels (4.5–10 μIU/L) aged between 18 and 50 years were randomly assigned to treatment (*n* = 25) or placebo (*n* = 25) groups for an 8-week treatment period.

Eight weeks of Ashwagandha treatment significantly improved serum TSH, T3, and T4 levels compared to placebo. Thus, it has been shown that treatment with Ashwagandha may be beneficial for normalising thyroid markers in patients with subclinical hypothyroidism [123].

### 3.13. Increase Muscle Strength

Ashwagandha supplementation has been shown to significantly increase muscle strength and to stimulate muscle renewal processes. In one study conducted, young healthy men were orally administered 300 mg of *Withania somnifera* root extract twice daily for eight weeks. These men also performed physical exercise—subjects participated in a structured resistance training program based on the publications of the National Strength and Conditioning Association (NSCA). A significant increase in muscle strength was observed in the treated patients, as well as an increase in muscle mass in the arms and chest. It was noted that in the Ashwagandha-supplemented patients, the level of exercise-induced muscle myocyte damage was significantly lower than in the placebo group, as indicated by the stabilisation of plasma creatine kinase levels. In addition, a significant increase in testosterone levels and a significant decrease in body fat were noted in the treated group [126]. Shenoy et al. [127], in their study, confirmed that the group receiving Ashwagandha supplementation had significant improvements in several measures of cardiorespiratory endurance compared to the placebo group. Specifically, the Ashwagandha group showed a significant increase in maximal aerobic capacity, time to exhaustion, and ventilatory threshold. Additionally, the Ashwagandha group had lower levels of serum cortisol, a hormone associated with stress. A study was also conducted on a group of adult athletes who were given a strictly defined dose of Ashwagandha, while the other group received a placebo. At the end of the study, a significant increase in VO_2_ max (maximum aerobic capacity) was observed in the treated group of athletes, compared to the placebo group. The athletes treated with Ashwagandha had significantly higher Total Quality Recovery Scores (TQR). An improvement in quality of life was observed in Ashwagandha-treated athletes (this was investigated based on results obtained from the DALDA—Daily Analysis of Life Demands for Athletes—questionnaire). It was estimated from the Recovery Stress Questionnaire (RESTQ) scores that treated athletes recovered more easily from exercise—they were less tired and had more energy—compared to the placebo group. A significant increase in antioxidant levels was also noted in the treated group. No adverse effects were observed throughout the study, indicating that this plant can be used safely [128]. In addition, an aqueous extract of *Withania somnifera* effectively increased muscle strength and induced fat growth. The study found that after 8 weeks of supplementation with Ashwagandha, the participants had significantly greater increases in muscle strength and power, compared to those who received a placebo. Additionally, the participants who took Ashwagandha had faster muscle strength recovery following a muscle-damaging exercise compared to the placebo group. [129].

### 3.14. Other Effects of Ashwagandha

Some medical studies suggest that Ashwagandha may also have applications in the treatment of COVID-19 as it exhibits many valuable properties such as the maintenance of immune homeostasis, regulation of inflammation, suppression of pro-inflammatory cytokines, protective effects on many organs, and anti-stress, anti-hypertensive, and anti-diabetic effects It may therefore be a valuable adjunct therapy for COVID-19 sufferers and may also have a beneficial effect on co-morbidities. However, more research is still needed. Studies indicate that witanoside V and somniferin, isolated from Ashwagandha, may be potential inhibitors of the major SARS-CoV-2M protease [130]. In addition, witanolides isolated from Ashwagandha appear to be valuable phytochemicals with antiviral activity in the context of COVID-19 treatment [131]. Withanone from Ashwagandha demonstrated antiviral activity in vitro by targeting the host’s major viral protease (MPro) and transmembrane TMPRSS2 [132]. All effects are summarized in Table 1.

## 4. Safety of Use

The long history of the medicinal use of Ashwagandha is primarily a confirmation of its efficacy and of its good tolerance by the body. Nevertheless, nowadays, many researchers are trying to eliminate concerns regarding its use. Recent reports of liver damage are worrying. Herbal supplements are a large and growing component of pharmacological markets, both nationally and internationally. Therefore, the monitoring of its safety is ever more important.

In 2004, the first case linking Ashwagandha to liver disease was discovered in Japan [137]. It concerned a 20-year-old man who had congestive liver damage and recovered without complications after withdrawal from Ashwagandha and 2 months of symptomatic treatment with ursodeoxycholic acid and phenobarbitone. Björnsson et al. [138] reported that Ashwagandha was the cause of five cases of liver damage. These cases illustrate the hepatotoxic potential of Ashwagandha. Liver damage is usually cholestatic or mixed with severe jaundice and pruritus, but is self-limiting, with normalisation of liver test results within 1–5 months. In addition, in the UK, a case was reported where a 39-year-old woman was diagnosed with jaundice and nausea after taking an over-the-counter herbal supplement containing Ashwagandha root extract [139]. There was also a report of a 41-year-old woman who, while taking Ashwagandha extract and progesterone, qualified for a liver transplant due to her deteriorating condition [140]. Reports of hepatotoxic effects are so far scarce and inconclusive. However, further reports should be monitored.

A study conducted in India on a group of 80 fully healthy individuals confirmed the lack of toxicity of this raw material. The participants were each administered 300 mg of Ashwagandha root extract orally, twice daily for 8 weeks. This was assessed by monitoring parameters such as body weight, systolic and diastolic blood pressure, haemoglobin, alkaline phosphatase, alanine transaminase, aspartate transaminase and plasma neutrophil and platelet counts. The values of the above indicators at the end of the study showed no significant differences between the group using the extract (40 subjects) and the group taking placebo (40 subjects). Thyroid function was also monitored by measuring blood levels of triiodothyronine, thyroxine and TSH; however, there were also no significant differences in the levels of these hormones [141].

Another study on a smaller group of volunteers (18 people) confirms, among other things, the lack of significant effects on red blood cell count, white blood cell percentage, ESR value, bilirubin, and plasma protein levels. However, an increase in serum creatinine and a decrease in blood urea nitrogen levels were observed. The researchers attributed this phenomenon to the concomitantly observed increase in muscle mass during the study. Volunteers took aqueous extracts over a 10-day period in doses that increased over time, starting with the equivalent of 6 g and ending with the equivalent of 10 g of vitania sluggard root [142]. Despite its many benefits, the plant should not be used during breastfeeding and pregnancy. Evidence is still lacking to unequivocally confirm the safety of taking Ashwagandha-based preparations during such sensitive periods of life. Some insight into this aspect of safety is provided by studies evaluating the effects of vitania sluggard extract on pregnant rats. The focus was primarily on the period between days 5 and 19 of pregnancy. Importantly, this is a particularly sensitive time due to increased organogenesis and histogenesis in the fetus. Doses were administered orally, the highest of which was 2000 mg/kg/day. No toxic effects were observed as a result of the study, and no changes were observed in the body weight of the pregnant females, the number of corpus luteum or embryo implantation. Furthermore, no external, skeletal or visceral deformities of the fetuses were detected [143].

## 5. Contraindications

Phytotherapy with Ashwagandha root is becoming more common, but it is important to note that the preparations are not recommended for all patients without controlling the other therapies they are receiving. Patients with hyperthyroidism experiencing symptoms such as irritability, restlessness, nervousness, anxiety, hand trembling, palpitations, psychomotor agitation, muscle weakness and fatigue, and decreased libido may be included in this group. Although preparations containing vitania sluggard root have proven efficacy in relieving the above-mentioned symptoms, their use in people with hyperthyroidism is contraindicated, as they exacerbate the effects of the disease. As studies show, this raw material increases the concentration of 3,3’,5-triiodothyronine (T3) and tetraiodothyronine (T4), which is unfavourable in hyperthyroidism [144].

Indian ginseng root extract has been used in the treatment of male infertility; however, men with hormone-sensitive prostate cancer should avoid using the raw material. According to studies, the plant can increase testosterone production [3], which intensifies the progression of the disease. The serum is absolutely contraindicated in patients planning or in pregnancy, as the use of higher doses of Ashwagandha root extract can lead to miscarriage [145].

Due to the reported effect of methanolic extract from Ashwagandha root on dopaminergic neurons in the ventral tegmental area via GABA-A receptors, extreme caution should be exercised when using sluggish vitania concomitantly with drugs acting via the indicated receptor (especially drugs from the benzodiazepine and barbiturate groups) for fear of potentiating the effects of the substances taken [146]. Ashwagandha root may interact with anti-anxiety, sleep, myorelaxant and sedative preparations, exacerbating their effects due to synergism. The raw material exhibits additive effects with anticonvulsants, barbiturates, and benzodiazepines which may lead to an increase in their adverse effects, such as impaired motor coordination, muscle weakness, headache, decreased libido, muscle tremors, and drowsiness [147].

Studies of Ashwagandha root extracts suggest that the raw material may be a CYP3A4 inducer or a CYP2B6 inhibitor [148], leading to clinically relevant raw material–drug interactions. As a direct consequence of this phenomenon, the adverse effects of the drug may be exacerbated, or the drug may be ineffective in the ongoing therapy. In addition, patients taking hypoglycaemic, hypotensive or immunosuppressive drugs, as well as those suffering from autoimmune diseases, should consult a doctor about possible Ashwagandha therapy. 

On the basis of available studies in humans and animals, it can be concluded that sluggard is a safe plant not only for short-term use, but also for long-term use. To date, no significant adverse effects have been shown to result from the ingestion of the raw material or its preparations. The main contraindications would be hypersensitivity reactions to plants of the Solanaceae family or, specifically, to this species [112,149].

In order to avoid autoimmunity from immune system supplementation, the raw material should be avoided by patients suffering from autoimmune diseases. The immunostimulant effect of Ashwagandha may aggravate the progression of conditions such as multiple sclerosis (MS), lupus (SLE systemic lupus erythematosus), and rheumatoid arthritis (RA). Therefore, preparations containing Ashwagandha root should not be used concomitantly with immunosuppressive drugs. The raw material and the drug exhibit antagonistic effects.

## 6. Conclusions

Ashwagandha is a plant material that has been used for centuries in traditional medicine systems, particularly in Ayurvedic medicine. Over the years, research has been conducted to investigate the various effects of Ashwagandha, and this research has shown that it has multiple beneficial effects on different body systems. However, it is important to note that research on Ashwagandha is ongoing, and more studies are needed to confirm its potential therapeutic uses and to determine the optimal doses and durations of use. Additionally, it is important to consider the safety of Ashwagandha, particularly when used in combination with other medications or supplements. Therefore, ongoing research, particularly clinical trials, is necessary to provide further insights into the potential benefits and risks of using Ashwagandha as a therapeutic agent. Based on research that has been carried out to date, it can be seen that Ashwagandha root is a plant raw material with multidirectional effects. However, due to the multitude of emerging reports, it is necessary to continuously update the knowledge on this raw material, both with regard to the possibility of its use in disease treatment and above all, with regard to its safe use. In addition, the determination of the effects of Ashwagandha requires ongoing research, mainly clinical, to confirm the efficacy of the raw material. The findings suggest that Ashwagandha may have therapeutic potential, especially for a range of neurological disorders. Although there is evidence to support the potential therapeutic uses of Ashwagandha, the mechanisms by which it exerts its effects are not yet fully understood. It is important to determine the precise mechanisms of action of Ashwagandha in order to develop more targeted and effective therapeutic strategies

## Figures and Tables

**Table 1:** Summary of the results of selected research studies on Ashwagandha *(Withania somnifera)*.

Disease	Target	Likely Mechanism of Action	Type of Study	Observed Activity	Reference
Alzheimer’s disease	The use of MTM to investigate how it affected the expression of the genes involved in neural plasticity and HDAC2 in an Alzheimer’s disease cell culture model.	An sp1 inhibitor called MTM prevented HDAC2 overexpression and resulted in much lower HDAC2 gene and protein expression, which restored the expression of synaptic plasticity genes in SH-APP cells.	In vitro model system	Inhibition of amyloid-beta production and activated B cells (NF-κB)-associated neuroinflammatory molecules’ gene expression—prevention of neuroinflammation and neurodegeneration	[10]
	Removal of toxic Aβ peptide in the brain	Decreased expression of RAGE and clusterin. Selective down-regulation of liver LRP and degradation of Aβ.	In vivo mouse model	Increased levels of sLRP in plasma, enhanced expression of LRP and NEP in the liver, concomitant increase in plasma Aβ42/40 and decrease in brain Aβ monomer levels—reversal of behavioural dysfunctions	[11,12]
	The ability of *Withania somnifera* derivatives to improve the fibril formation of amyloid-β 42 in Alzheimer’s disease	Interaction with the hydrophobic core of amyloid-β 1–42 during the oligomeric stage, thus inhibiting further interaction with the monomers and reduces aggregation	In vitro studies	Decrease in apoptotic cells and reactive oxygen species.	[15]
	Neuroprotective effect of Withaferin A	Cell toxicity signaling pathway inhibition involving PI3K/mtor pathway	In vitro studies	Protection of dopaminergic and cortical neurons. Increase in cell survivor.	[17]
	Withanolide A’s ability to penetrate the brain and protect against cerebral ischemia-reperfusion damage	Inhibition of matrix metalloproteinases-2 (MMP-2). Lowered elevated levels of glutamate and GABA. However, more studies required to properly elucidate the mechanism of action.	In vivo mouse model	Decrease in apoptotic and necrotic cell death. Reduction in morphological damage of brain tissues. Reduced cerebral infarction and oedema. Restored blood-brain barrier disruption.	[18]
Parkinson’s disease	Protection of neuronal injury in Parkinson’s disease and physiological abnormalities	Upregulation Of DA receptors after lesioning. Use of gpx which uses H202 to oxidize GSH in order to defend against hydrogen peroxide toxicity and detoxify free radicals and lipid peroxides. Further studies required for description of mechanism especially for dosage form. Interference with oxidative damage.	In vivo mouse model	Reversal of toxic effects of 6-OHDA, muscle, and locomotive activity. Increase in striatal content and dopaminergic D2 receptor populations in striatum. Improvement of enzyme activity hence physiological functions	[22,26]
	*W. somnifera* root extract, protective effects against 6-OHDA-induced toxicity in the human neuroblastoma SH-SY5Y cell line	Increase in glutathione peroxidase activity and thioltransferase activity. Modulation of oxidative response proteins and the control of redox regulation via S-glutathionylation	In vitro cell lines	Increase in ATP levels and decrease in protein-glutathionylation levels in the cells.	[23]
	Effect of *W. somnifera* on catecholamines and physiological abnormalities	Induction of catecholamines, antioxidants, and translation of proteins hence Cell growth	In vivo mouse model	Increased DA, DOPAC and HVA levels and normalized TBARS levels in the corpus striatum. Improved motor function	[24]
	Evaluation of the neuroprotective effects of *W. somnifera* extract on the LRRK2 loss-of-function	Decrease in PSP amplitude and suppression of mutation by reversal of mutation-related loss of mitochondrial structural integrity	In vivo Drosophila melanogaster model	Improved motor and muscle activity. Protection against mitochondria degeneration	[25]
Huntington’s disease	Restoration of biochemical alterations caused by 3-NP	Not yet fully understood and require further research	In vivo mouse model	Restoration of mitochondrial enzyme activity and antioxidant enzymes in striatum and cortex of the brain, reversal of muscle impairment, reduction of lipid peroxidation, nitrate, and dehydrogenase enzymes	[28]
	Elongation of lifespan with administration of Withaferin A	Activation of HSF1 and induction of HSR chaperones	In vivo mouse model	Decrease in inflammatory process and mutant huntingtin aggregates. Improvement of striatal function	[29]
Treatment of obsessive-compulsive disorder, alcohol withdrawal syndrome	Alleviating symptoms of obsessive-compulsive disorder	Not yet fully understood and require further research—likely impact on the serotonin system	Randomized double-blind placebo-controlled trial	Significantly greater effect of W. somnifera in alleviation severity of OCD assessed using Yale-Brown Obsessive-Compulsive Scale (Y-BOCS) in a randomized double-blind placebo-controlled trial	[35]
			In vivo rat model	Decrease in marble hiding behaviour activity behavioural activity without affecting motor activity in rats	[36]
			In vivo rat model	Beneficial effects on controlling behavioural changes, anxiety and seizures in alcohol withdrawal symptoms in rats, and improve locomotor activity.	[38]
			In vivo rat model	Alleviation of withdrawal anxiety due to chronic alcohol consumption,	[37]
Anti-inflammatory/immunomodulatory effects mi	Alleviating inflammation and improving immunity	Not yet fully understood and require further research—likely Inhibition of lipopolysaccharide (LPS)-activated NFκB, P38 and JNK/SAPK MAPK pathways, regulating cytokines	In vivo mouse model	Inhibition of the NF-κB and MAPK (mitogen-activated protein kinase) pathways by de-creasing the expression of pro-inflammatory cytokines, including interleukin (IL)-8, IL-6, tumour necrosis factor (TNF-α), IL-1β, and IL-12, and increasing the expression of anti-inflammatory cytokines	[41]
			In vivo mouse model	Potent inhibitory effect on proteinuria, nephritis, and other inflammatory markers such as cytokines including interleukin (IL)-6 and tumour necrosis factor (TNF)-α, nitric oxide (NO), and ROS in a mouse model of lupus	[133]
			In vivo rat model	Changes in the concentrations of a number of serum proteins such as α2 glycoprotein, acute phase protein α1 and prealbumin were demonstrated, along with a significant reduction in inflammation	[40]
			In vivo mouse model	Increase in the total number of white blood cells and bone marrow cells, as well as to increase the titre of circulating antibodies and antibody-producing cells, and to stimulate the production of immune cells and phagocytosis of macrophages	[44]
			In vivo rat model	Changes in the concentrations of serum proteins such as α2 glycoprotein, acute phase protein α1 and prealbumin with a significant reduction in inflammation	[40]
			In vivo rat model	Inhibition of reactive gliosis, production of inflammatory cytokines such as TNF-α, IL-1β, IL-6, and expression of nitrooxidative stress enzymes	[60]
			Randomized double-blind placebo-controlled trial	Significantly increased natural killer cell activity and cytokine levels compared to placebo	[45]
			Randomized double-blind placebo-controlled trial	Significant increase in natural killer cell activity and cytokine levels in a randomized, double-blind, placebo-controlled trial	[45]
Antibacterial properties	Inhibiting the growth pathogenic bacteria	Not yet fully understood and require further research–likely disrupting bacterial cell membranes—more research needed	In vitro	Inhibition of the growth of methicillin-resistant *Staphylococcus aureus* and *Enterococcus* spp.	[46]
			In vitro	Effective inhibition the growth of *Staphylococcus aureus*, *Proteus mirabilis, Escherichia coli* and *Pseudomonas aeruginosa.*	[122]
			In vitro	Effective inhibition the growth of Escherichia coli, Salmonella typhi, Citrobacter freundii, Pseudomonas aeruginosa and Klebsiella pneumoniae.	[49]
			In vitro	Effective treatment for salmonellosis, as it significantly alleviates the course of infection following infection with this pathogen	[51]
			In vitro	Significantly slowness the growth of bacteria present in the oral cavity, such as *Streptococcus mutant* and *Streptococcus sobrinus*	[52]
			In vitro	Induction of cell death (acts on promastigotes) of *Leishamania donovani* by activating the process of apoptosis	[53]
			In vitro	Antifungal properties against some fungal species; it inhibits *Candida albicans*	[48]
			In vitro	Antifungal properties *of Withania somnifera* glycoprotein from its root tubers, against *Aspergillus flavus*, *Fusarium oxysporum*, *Fusarium verticilloides,* and antibacterial properties against *Clavibacter michiganensis* subsp. michiganensis	[54]
			In vivo mouse model	Effective in the treatment of malaria, significantly reducing parasitaemia	[56]
Support for infertility treatment	Improve the quality of semen	Not yet fully understood and require further research—likely effect on the GABA receptors, thus facilitating the expression of GnRH expression; structural similarity to testosterone and thus imparted the benefits of male steroidal hormones; regulation of oxidative stress	In vivo study	Decrease in stress, improved the level of antioxidants and improved overall semen quality	[58]
			Randomized, double-blind, placebo-controlled study	Statistically significant increase in the total DISF-M (the derogatis interview for sexual functioning-male) scores	[65]
			Triple-blind randomised clinical trial	Increased mean sperm count and progressive motility and improved sperm morphology compared to the baseline	[59]
			Clinical trial	Repair of disturbed plasma concentrations of lactate, alanine, citrate, GPC, histidine and phenylalanine and restores semen quality	[60]
			A randomized controlled trial	Significant subjective perception of sexual well-being and assisted in increasing serum testosterone levels in the participants.	[65]
Anticancer effects	Inhibition of cancer cell proliferation	Not yet fully understood and require further research	In vitro	Activation by withaferin A the TRIM16 protein, leads to the degradation of cancer-related proteins and ultimately induces cell death in melanoma cells	[68]
			In vitro	Effectiveness of withaferin a in the treatment of melanoma by compound induces apoptosis reduction cell proliferation and inhibits melano-ma cell migration	[69]
			In vitro/In vivo	Inhibited GBM growth in vitro and In vivo and triggered intrinsic apoptosis of GBM cells	[70]
			In vitro	Inhibition of proliferation of human endometrial cancer cells by Withaferin A through the modulation of TGF-β signaling and the inhibition of TGF-β dependent Smad2 phosphorylation	[134]
			In vitro	Withaferin A alone or in combination with standard chemotherapy is a potential treatment option for EGFR (epidermal growth factor receptor) wild-type lung cancer and may decrease the occurrence of cisplatin resistance by inhibiting lung CSCs (cancer stem-like cell).	[135]
			In vitro/In vivo mouse model	combination of extract and intermittent fasting decrease cancer cell proliferation through apoptosis induction, while also reducing cisplatin-induced toxicity in the liver and kidney.	[71]
			In vivo rat model	protective effect against acute and chronic gamma radiation-induced damage to the liver and spleen tissues of rats	[72]
Antidiabetic activity	Lowering blood sugar levels	Not yet fully understood and require further research—likely Improving insulin sensitivity, stimulating beta cells, reducing inflammation, and protecting against oxidative stress, inhibition of α-glucosidase	In vivo rat model	Decrease in fasting blood glucose levels in rats with STZ-induced hyperglycaemia	[73]
			In vivo rat model	Improvement diabetes-induced testicular dysfunction in pre-adolescent rats.	[74]
			In vivo rat model	Efficacy against elevated plasma glucose, insulin and cortisol levels and changes in adrenal and spleen weights in diabetic animals	[80]
			Double-blind randomized control trial	Improve antioxidant parameters and lipid profile, and demonstrate the tolerability and safety	[84]
Treatment of sleep disorders	Improve the quality and length of sleep	Not yet fully understood and require further research—likely effect on GABAergic activity	In vivo mouse model	Significant induction of NREM (Non-Rapid Eye Movement) sleep in research on mice	[93]
			In vivo rat model	significant reduction in the levels of free radicals, lipid peroxidation, and an increase in the levels of antioxidant enzymes in the sleep-deprived rat group	[102]
			Randomized, double-blind, placebo-controlled study	Improvement in the general wellbeing, sleep quality, and mental alertness in a prospective, randomized, double-blind, placebo-controlled study	[92]
			Randomized, double-blind, placebo-controlled study	Significantly improved the quality of sleep and easier and faster to falling asleep	[84]
			Randomized, double-blind, placebo-controlled study	Sleep efficiency, sleep duration and total sleep time improvements in physical, psychological, and environmental areas were also noted	[98]
Cardioprotective properties	Protection of heart cells against harmful agents	Not yet fully understood and require further research—likely anti-apoptotic properties due to an increase in AMP-activated protein kinase (AMPK) phosphorylation and an increase in the Bcl-2/Bax ratio (AMPK) and by restoring oxidative balance	In vivo rat model	A decrease in glutathione levels, a decrease in the activity of superoxide dismutase, catalase, creatinine phosphokinase, and lactate dehydrogenase albino rats in which myocardial necrosis treated with Withania Somnifera.	[85]
			In vivo rat model	Reduction of the damage to the heart caused by ischemia induced in rats	[86]
			In vivo rat model	In this study in rats, low doses of withaferin A were shown to have a cardioprotective effect by upregulating the mitochondrial anti-apoptotic pathway due to an increase in AMP-activated protein kinase (AMPK) phosphorylation and an increase in the Bcl-2/Bax ratio (AMPK).	[87]
Anxiolytic and anti-stress effects	Calming and stress-relieving effect	Not yet fully understood and require further research—likely moderating effect on the hypothalamus-pituitary-adrenal axis (HPA); antioxidant and anti-inflammatory effects	A randomized, double-blind, placebo-controlled study	Reduction in the HAM-A (Hamilton Anxiety Rating Scale) IN A randomized, double-blind, placebo-controlled study	[103]
			A double-blind, randomized, placebo-controlled clinical study	Reduction in PSS (perceived stress scale) scores I A Double-blind, Randomized, Placebo-controlled Clinical Study	[108]
			A randomized, double-blind, placebo-controlled study	A reduction in stress levels, improvement in memory and attention, sleep quality, and overall psychological well-being.	[112]
			Randomized double-blind placebo-controlled trial	Potentially support SSRI therapy in patients diagnosed with GAD syndrome	[107]
			Randomized, placebo-controlled clinical trial	Medium effect sizes over placebo for depression single-item and anxiety-depression cluster scores. Adverse events were mild and transient	[114]
			Double-blind randomized control trial	Increased of college students’ perceived well-being through supporting sustained energy, heightened mental clarity, and enhanced sleep quality	[104]
			Double-blind randomized control trial	Improvements in attention and working memory, as well as reductions in symptoms of anxiety and stress, as a result of Withania somnifera supplementation in adults	[119]
Adaptogenic effect	ability to adapt and maintain homeostasis in response to various stressors, both physical and emotional	Not yet fully understood and require further research—likely regulation of the HPA axis, antioxidant effects, immunomodulatory effects, and modulation of neurotransmitter signaling.	In vivo equine model	Adaptogenic and immunomodulatory activity in an equine model, potentially improving the health and performance of horses	[136]
			In vivo rat model	Significant anti-stress effects, including the modulation of the HPA axis, increased levels of antioxidant enzymes, and reduced levels of lipid peroxidation,	[121]
Hypothyroidism	Increase in the concentration of thyroid hormones	Not yet fully understood and require further research	A double-blind, randomized placebo-controlled trial	Effective normalisation of serum thyroid indices during the 8-week treatment period	[123]
			In vivo rat model	Significant parameter improvements in serum TSH level, serum glucose, Il-6, body weight gain, hepatic and renal MDA and NO, the values of GSH, gpx and Na+/K+-atpase, and improvement in thyroid histology	[124]
			In vivo mouse model	Significant reduction of hepatic lipid peroxidation, whereas the activity of antioxidant enzymes such as superoxide dismutase and catalase were increased	[125]
Increase muscle strength		Not yet fully understood and require further research, likely increase in testosterone levels, reductions in muscle damage and inflammation, and antioxidant and anti-inflammatory effects.	A double-blind, randomized placebo-controlled trial	Ashwagandha supplementation association with increased muscle strength and endurance in older adults who performed resistance training.	[126]
			A double-blind, randomized placebo-controlled trial	Increase in cardiorespiratory endurance and an improvement in quality of life	[128]
			A double-blind, randomized placebo-controlled trial	Extract of Ashwagandha on muscle strength, power, and recovery in healthy men who engaged in resistance training.	[129]
			A double-blind, randomized placebo-controlled trial	Significant improvements in cardiorespiratory endurance measures of the elite Indian cyclists	[127]

## Data Availability

Not applicable.
